# Simulation of laser-induced tunnel ionization based on a curved waveguide

**DOI:** 10.1038/s41598-023-39142-1

**Published:** 2023-08-03

**Authors:** Arnon Ben Levy, Amir Hen, Merav Kahn, Yoad Aharon, Tamar Levin, Noa Mazurski, Uriel Levy, Gilad Marcus

**Affiliations:** Institute of Applied Physics, Hebrew University of Jerusalem, 91904 Jerusalem, Israel

**Keywords:** Attosecond science, Imaging and sensing

## Abstract

The problem of tunneling ionization and the associated questions of how long it takes for an electron to tunnel through the barrier, and what the tunneling rate has fascinated scientists for almost a century. In strong field physics, tunnel ionization plays an important role, and accurate knowledge of the time-dependent tunnel rate is of paramount importance. The Keldysh theory and other more advanced related theories are often used, but their accuracy is still controversial. In previous work, we suggested using a curved waveguide as a quantum simulator to simulate the tunnel ionization process. Here we implemented for the first time such a curved waveguide and observed the simulated tunneling ionization process. We compare our results with the theory.

## Introduction

At laser intensities above ${10}^{12} \text{W/c}{\text{m}}^{2}$ , strong field interaction with atoms, molecules, and solids brings in a rich set of physical phenomena associated with attosecond physics. Processes such as high harmonic generation(HHG)^1–4^, Above threshold Ionization (ATI)^5,6^, laser-induced electron diffraction (LIED) imaging^7–9^, laser-induced inner-shell excitation^10–12^ and steering electrons in atoms and molecules^13,14^. At such intensities, the radiation’s electric field becomes comparable to the field that the electron feels inside atoms and molecules, hence, perturbative approaches to understanding these processes are not valid. One of the most fruitful approaches to gaining insight into the physics of such interactions is the semiclassical three-step model^15,16^. In the first step of this model, the strong laser field deforms the Coulomb potential and creates a potential barrier, through which the electron can tunnel out and appears at the continuum as an ionized electron. The two other steps are acceleration of the electron in the laser field and recollision with the parent ion with an excess of energy. In this semiclassical model, the tunnelling step is a quantum phenomenon that has no counterpart in classical mechanics. For this reason, we also lack an intuition about this process and many questions about it remain open with controversial answers. Relevant to our case are the questions of how long it takes for the particle to traverse the potential barrier? what is the particle velocity under the barrier? and what is the particle velocity exactly at the exit point from the barrier? Understanding the tunnelling process is essential for predicting and controlling related recollision phenomena such as HHG, ATI, and LIED. For example, the above-mentioned three-step model assumes that the electron emerges at the exit of the potential barrier with zero velocity. This zero velocity is then used as the initial condition for the classical calculation of the electron trajectories under the action of the electromagnetic field. This assumption is often used to understand and interpret the results of many experiments where laser-induced electron recollision is involved such as two-color HHG spectroscopy^17–19^, non-sequential double ionization^20,21^, inelastic core–hole excitations^10–12^, Sub-cycle electron control in the photoionization^13,14^, and laser field streaking.

The question of how long it takes for a particle to tunnel and traverse under a static barrier has fascinated scientists since 1931 when Condon^22^, and MacColl^23^ a year after, first raised this problem. In the 1960s, when tunneling became relevant for technology, Hartman revisited this question and used the method of stationary phase to calculate the group delay between the incoming wave packet and the outgoing wave packet. His analytical calculation suggests a group delay that saturates with increasing barrier thickness which implies a superluminal velocity beyond a certain barrier thickness^24^. An alternative approach was proposed by Baz’ for measuring the delay by using a quantum–mechanical clock based on the Larmor precession of a spin in the presence of a magnetic field^25^. Nevertheless, the limitations raised by Hartman, persist also with the Larmor clock approach^26^. Over time, various definitions for tunneling time were proposed, but their interpretation stays controversial. For an excellent review on this subject see^27^.

In the context of tunneling ionization from atoms, it was L. D. Landau and E. M. Lifshitz who solved analytically this problem for the hydrogen atom under the static electric field and found the ionization rate to be:

1 $$\varpi =4{\varpi }_{a}\frac{{E}_{a}}{\left|E\right|} {\text{exp}}\left[-\frac{2}{3}\frac{{E}_{a}}{\left|E\right|}\right]$$

where $${E}_{a}=\frac{{m}^{2}{e}^{5}}{{\hslash }^{4}}= \frac{2Ry}{e{a}_{0}}, {\varpi }_{a}=\frac{m{e}^{4}}{{\hslash }^{3}}= \frac{2Ry}{\hslash }$$, $$Ry=13.6\text{eV, }{a}_{0}=\text{Bohr radius=53pm}$$.

In 1964, L.V. Keldysh calculated the transition rate of an electron from the atomic ground state to the Volkov states^28^ of the free electron under the action of a strong oscillatory electric field^29^. He obtained a general solution with two limiting cases which are separated by the adiabaticity parameter $\Gamma =\omega /{\omega }_{t}$ where $\omega$ is the oscillation angular velocity and ${\omega }_{t}=eE/\sqrt{2m{I}_{p}}$ is a characteristic rate where ${I}_{p}$ is the ionization potential $e$- the electron charge, and $E$- the electric field. The adiabaticity parameter, known also as Keldysh parameter, is often given as $\Gamma =\sqrt{{I}_{p}/2{U}_{p}}$ where ${U}_{p}={e}^{2}{E}^{2}/4m{\omega }^{2}$ is the average kinetic energy of a free electron under the action of oscillatory field and is known as the ponderomotive energy. In the case of high frequency ($\Gamma \gg 1$) this expression describes a multiphoton ionization rate, corresponding to the ATI. In the low-frequency limit ($\Gamma \ll 1$) he obtained:

2 $$\varpi =\frac{\sqrt{6\pi }}{4}\frac{{I}_{p}}{\hslash } {\left(\frac{\hslash \omega }{{I}_{p}} \sqrt{\frac{{e}^{2}{E}^{2}}{m{\omega }^{2}{I}_{p}}}\right)}^\frac{1}{2}{\text{exp}}\left\{-\frac{4}{3}\frac{{I}_{p}}{\hslash \omega } \sqrt{\frac{2m{\omega }^{2}{I}_{p}}{{e}^{2}{E}^{2}}}\left(1-\frac{m{\omega }^{2}{I}_{p}}{5{e}^{2}{E}^{2}}\right)\right\}$$

As $\omega \to 0$, the exponential in (2) coincides with the obtained exponential term by Landau for the static tunneling ionization. Therefore, in the adiabatic limit, the instantaneous ionization rate follows the static tunneling ionization rate (Eq. (1)) with the instantaneous electric field as a parameter. It is worth noting that the electric field in the pre-exponential term of Eq. (2) has a power of one half while in the Landau tunnel ionization, it has a power of minus one. Equation (2) was calculated for ionization of hydrogen atom from the ground state. Later, it was extended by Faisal^30^ and Reiss^31^ (KFR theory) to obtain the ATI spectrum, by Perelomov, Popov, and Terent’ev^32^ (PPT theory) and by Ammosov, Delone, and Krainov^33^ (ADK theory) to include other atoms and other states with higher angular momentum. In PPT and ADK theories they have a factor of $\sqrt{3E/\pi {E}_{a}}$ multiplying the Landau term (Eq. 1), due to the assumption of slow envelope and averaging the ionization rate over one laser oscillation period^32^.

With the advent of picosecond and later femtosecond lasers a series of experiments were carried out to test the ionization yield with respect to the laser intensity^34–36^. These experiments confirmed the transition from perturbative multiphoton ionization to non-perturbative tunneling ionization with a rough agreement with the above-mentioned theories, but sometimes with deviations of order of magnitude and more. Some of the deviations were explained by the non-sequential double ionization process, but not all. Besides, such experiments test the integrated ion yield, accumulated during the whole pulse duration, but not the instantaneous ionization rate. Surprisingly there are only a few experiments to test directly the instantaneous tunneling ionization rate in the linear polarization case^37,38^. In parallel to those experiments, another set of experiments was set to determine the delay between the maximum of the laser electric field and the time at which the electron emerges at the tunneling exit. In that context, the so-called attoclock technique in which the tunneled electron is angularly streaked in a circularly polarized laser^39^, however, no clear conclusion came from these experiments: some claimed a zero time delay^40,41^ while others claimed a finite delay with values in the range of 30–100 attosecond^42,43^. HHG spectroscopy was used to answer the question of when the electron appears in the continuum^17^. Excellent review on tunneling time and what could or couldn’t be measured with attoclock is given in^44^ and reference therein.

## Quantum simulators

As mentioned above, the tunnel ionization process still contains many unresolved questions. Reasons for that are unintuitive or ambiguous definitions of tunneling time and the highly nonlinear nature of this process with the need to compromise with various approximations. From the experimental point of view, the lack of our ability to probe the electron inside the “under-the-barrier” region, forces us to extract this information indirectly from the electron spectra away from the atom or from the HHG spectra. In most cases, such an information extraction is model-dependent, therefore, contains all the model flaws. Quantum simulators might give us the ability to probe the “electron” wavefunction in all regions, including the ”under-the -barrier” region, and to extract from it physical observables such as probability density current, particle momentum, kinetic energy etc. . Cold-trapped atoms were proposed as a quantum simulator to probe the dynamics of atoms under the action of strong fields^45–49^. Another approach is to use quantum optical simulator. Since both quantum mechanics and electromagnetism are wave theories, they share many similarities. The longer wavelength of visible light compared to matter waves allows us to easily observe related phenomena in optics that are difficult to observe in matter waves. The immediate example is the two-slit interference pattern that is often demonstrated in high schools and undergraduate student laboratories, but is much more difficult to observe with matter waves. Nowadays, quantum-optical analogies are used to mimic many quantum systems and quantum phenomena in order to gain more insight. The interested reader may find an excellent review on the field of quantum optical simulators here^50^ and references therein. Recently, we proposed to use a curved optical waveguide to simulate the tunneling ionization process^51^. In this paper we present our first results from such a simulator.

**Figure 1:**
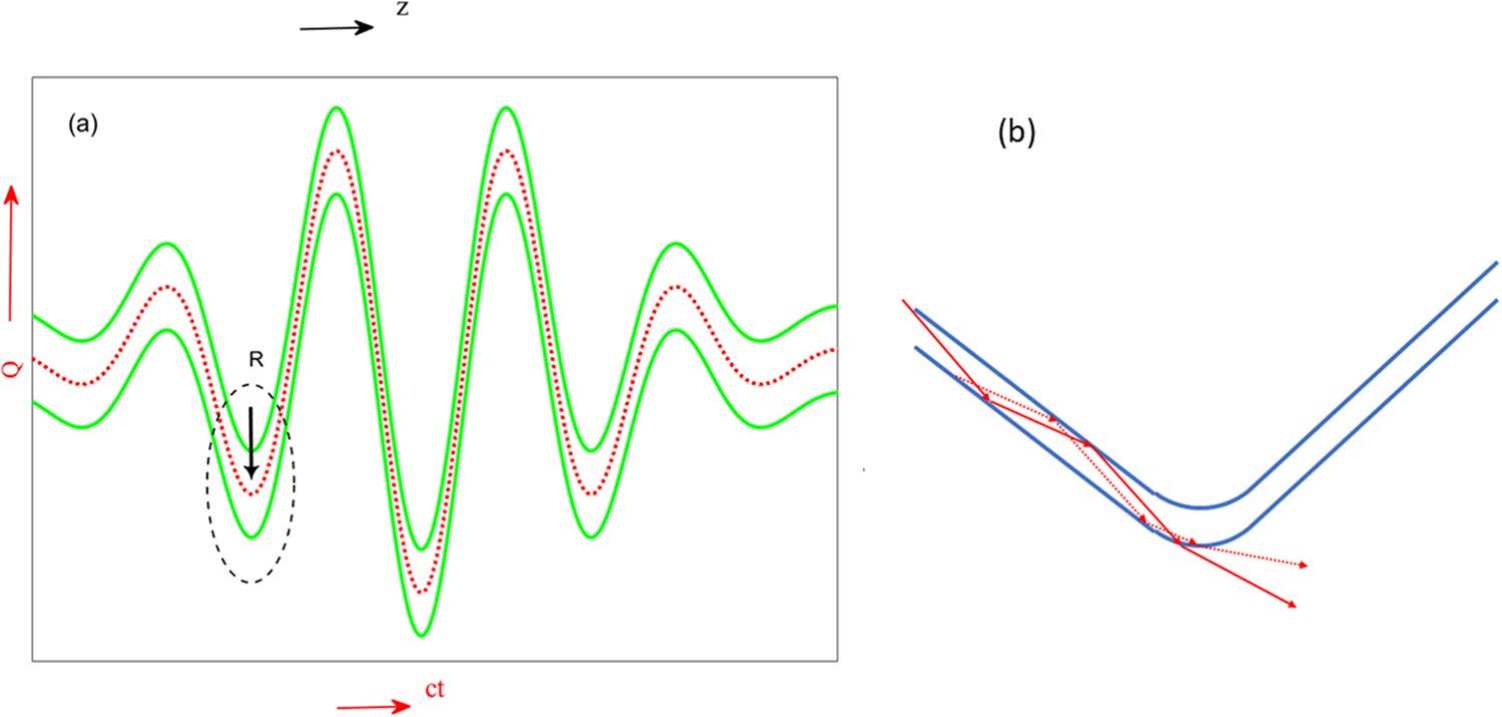
(**a**) shows the finite square well potential in the transformed coordinates $\widetilde{Q} (\widetilde{y})$ as a function of $\widetilde{t} (\widetilde{z})$. Red dotted line indicates quiver motion of the electron $q(t)$ as given in Eq. 4c. The green lines show the curved waveguide boundaries. (**b**) Zoom in on the black dotted ellipse of Fig. 1(**a**). Two optical rays hit the waveguide walls at an angle that is larger than the critical angle and experience total internal reflection. Because of the bending, at some point, the incidence angle goes below the critical angle and the rays may escape the waveguide according to Snell low.

It is well known that within the paraxial approximation, wave propagation in an optical dielectric waveguide is governed by a Schrödinger -like equation:

3 $$2i{k}_{cl}\frac{\partial E}{\partial z}=-{\nabla }_{T}^{2}E-2\frac{{\omega }^{2}}{{c}^{2}}{n}_{cl}\left(n(x,y)-{n}_{cl}\right)E$$

where ${n}_{cl}$ is the clad refractive index, ${k}_{cl}=\omega {n}_{cl}/c$ , and ${\nabla }_{T}^{2}=\frac{{\partial }^{2}}{\partial {x}^{2}}+\frac{{\partial }^{2}}{\partial {y}^{2}}$. Here, $\left(n(x,y)-{n}_{cl}\right)/{n}_{cl}$ plays the role of potential energy in the Schrödinger equation. To also include the interaction of the atom with the oscillating electric field $E={E}_{0}\mathrm{cos}\left(\omega t\right)$ in this quantum simulator, we make the following canonical transformation:

4a $$Q=x+q(t)$$

4b $$\Pi =p-\dot{q}(t)$$

4c $$\dot{q}\left( t \right) = \frac{e}{m}\smallint E\left( {t^{\prime } } \right){\text{d}}t^{\prime } = - \frac{e}{mc}A\left( t \right)$$

where $A(t)$ is the vector potential. Under this canonical transformation, the Hamiltonian $H=\frac{{p}^{2}}{2m}+V\left(x\right)-exE(t)$ become the transformed Hamiltonian $\widetilde{H}=\frac{{\Pi }^{2}}{2m}+V(Q-q\left(t\right))$. This coordinate transformation is well known as the Kramers-Henneberger transformation^52^.This transform Hamiltonian describes a particle that is placed in the same potential as in the original Hamiltonian, but now is shaken in time following the motion of $q\left( t \right) = - \frac{e}{mc}\smallint A\left( {t^{\prime } } \right)dt^{\prime }$.It is worth noting that the solution for the Schrödinger equation in the transformed coordinates for the case of a free electron, immediately yields the Volkov states^28^. We next examine the advantage of using the transformed coordinates. Figure 1a shows a finite square well potential in the new coordinates (Eq. (4)) as a function of time. If we take the above-mentioned analogy between a square well potential and a step-index waveguide, Fig. 1a also describes a curved waveguide. Such a bent waveguide may experience higher losses at high curvature points (Fig. 1b). The advantage of moving to the new coordinates is now clear: it allows us to extend the analogy between atoms and waveguides, and incorporate the coupling to the strong external field by imprinting it into the geometry of the waveguide. To put the time-dependent potential and the z-dependent refractive index on an equal footing we use dimensionless time and space coordinates as follows:

5 $$\begin{aligned} \widetilde{t} = & \left( {\frac{{mc^{2} }}{\hbar }} \right)t \\ \widetilde{Q} = & \left( {mc/\hbar } \right)Q \\ \widetilde{z} = & k_{{cl}} z \\ \widetilde{y} = & k_{{cl}} y \\ \end{aligned}$$

In^51^ we showed the correspondence between the Keldysh tunnel Ionization and the bending losses from curved optical waveguide. Briefly, the bending losses formula, derived by Marcuse^53^ is :

6 $${\varpi }_{BL}\left(R\right)=\frac{\sqrt{\pi }}{2}\frac{{\kappa }^{2} \gamma a {\text{ exp}}\left\{2\gamma a\right\}}{{V}^{2}\beta } {\left(\frac{{\beta }^{2}}{{\gamma }^{3}R}\right)}^{1/2}{\text{exp}}\left\{-\frac{2}{3}\frac{{\gamma }^{3}}{{\beta }^{2}}R\right\}$$

where $\kappa$ and $\gamma$ are the transverse wavevectors inside and outside the waveguide respectively, $a$ is the waveguide width and ${V}^{2}={\left(\frac{\omega a}{c}\right)}^{2}\sqrt{{n}_{co}^{2}-{n}_{cl}^{2}}$ . $R$ is the radius of curvature of the bent waveguide. At the same time, the Keldysh ionization rate (Eq. 2) in the limit of $\omega \to 0$ is:

7 $${\varpi }_{K}\left(\Gamma \right)=\frac{\sqrt{3\pi \sqrt{2}}}{2}\left(\frac{{I}_{p}}{\hslash }\right) {\left(\left(\frac{\hslash \omega }{{2I}_{p}}\right)\frac{1}{\Gamma }\right)}^\frac{1}{2}{\text{exp}}\left\{-\frac{2}{3}\left(\frac{{2I}_{p}}{\hslash \omega }\right)\Gamma \right\}$$

This limit is justified whenever $\omega <{\omega }_{t}=eE/\sqrt{2m{I}_{p}}$, i.e., when $\Gamma <1$ , but not too close to the electrostatic case, in which case the Landau ionization rate applies (Eq. (1)) ^29^.

Comparing the Keldysh ionization rate (Eq. (7)) with the bending loss rate (Eq. (6)), they look similar with $\left(\frac{{\gamma }^{3}}{{\beta }^{2}}R\right)$ in Eq. (6) plays the role of $\left(\frac{2{I}_{p}}{\hslash \omega }\Gamma \right)$ in Eq. (7). In^51^ we showed that if we take the radius of curvature definition $R\equiv \frac{{\left({\dot{z}}^{2}+{\dot{y}}^{2}\right)}^{1.5}}{\left|\dot{z}\ddot{y}+\dot{y}\ddot{z}\right|}$ where z and y are given as a function of a parameter t, and apply it also to the shaken potential coordinates $\left\{ct , q(t)\right\}$ we have

8 $$R\Leftrightarrow \frac{{\left[{c}^{2}+\frac{{e}^{2}}{{m}^{2}{c}^{2}}{A}^{2}\left(t\right)\right]}^{1.5}}{\frac{ceE\left(t\right)}{m}}$$

which in the nonrelativistic case converges to $\frac{m{c}^{2}}{eE}$. Under this approximation, it can be shown that $\left(\frac{{\gamma }^{3}}{{\beta }^{2}}R\right)$ indeed equal to $\left(\frac{2{I}_{p}}{\hslash \omega }\Gamma \right)$ and the radius of curvature in bending losses plays the same role as the Keldysh parameter in tunnel ionization. However, besides this striking resemblance, there is a conceptual difference between the two. While the Keldysh parameter depends only on the instantaneous electric field, in the $R$ parameter we have both the instantaneous electric field and the integration of it over its whole history: $A\left(t\right)=-c\underset{-\infty }{\overset{t}{\int }}E\left({t}{\prime}\right) dt{\prime}$. Again, this additional term is expected to play a significant role only in relativistic fields.

Here we fabricated a curved waveguide, observed the radiation leakages from it, and quantified the bending losses. The waveguide is fabricated on a ${\text{Si}}$ substrate. A layer of $2\mathrm{\mu m}$ of ${\text{Si}}{\text{O}}_{2}$ is grown on top of the ${\text{Si}}$, and a layer of $0.4\mathrm{\mu m}$ of ${\text{S}}{\text{i}}_{3}{{\text{N}}}_{4}$ is grown on top of the ${\text{Si}}{\text{O}}_{2}$. Next, we put a photoresist layer, imprinted the curved waveguide on it, and etched an 11nm layer from the $S{i}_{3}{N}_{4}$, leaving an 11nm rib waveguide. Finally, we caped the whole wafer with additional $2\;\mu {\text{m}}$ thick layer of ${\text{Si}}{\text{O}}_{2}$. The waveguide is $3\mu m$ wide and its curve is defined by the equation $y(z)={q}_{0} {\text{cos}}\left(2\pi z/\Lambda \right)\text{ exp}\left\{-{\left(z-{z}_{0}\right)}^{2}/{X}^{2}\right\}$ with $\Lambda =1400\mu m$ and $X=2100\mu m$. We fabricated a few waveguides with different amplitudes ranging from ${q}_{0}=40\mu m$ to ${q}_{0}=120\mu m$. We used a single-mode fiber-coupled laser diode at a wavelength of 785nm, expanded the beam with a telescope and a biprism and then coupled it into the waveguide with a microscope objective lens. The guided mode and the leakage from the waveguide were scattered from material imperfections and were observed from the top by a microscope. We use the effective index theory^54^ to calculate the waveguide refractive indices for the core and cladding. For the $T{E}_{\mathrm{0,0}}$ fundamental mode we found ${n}_{co}=1.9353, {n}_{cl}=1.9107,\Delta n=0.0042.$ Taking these parameters and using the analogy to a square well potential, we find that the case of $T{E}_{\mathrm{0,0}}$ mode is equivalent to a square well potential with a depth of ${V}_{0}=1125eV$, ${\text{I}}_{\text{p}}= \text{713eV}$, and a "driving laser" the with a central wavelength of 8.23 nm ("photon energy" of 150eV). Such values are not representative values for conventional tunneling ionization experiments. However, in^51^ we showed that we can use an additional scaling parameter S such that $z\to Sz, y\to \sqrt{S}y,\Delta n\to \frac{\Delta n}{S}$. Under this scaling and with S = 100 we got ${V}_{0}=11.25eV$, ${\text{I}}_{\text{p}}\text{=7.13eV}$, and the "driving laser" wavelength is 823nm ("photon energy" of 1.5eV).

**Figure 2:**
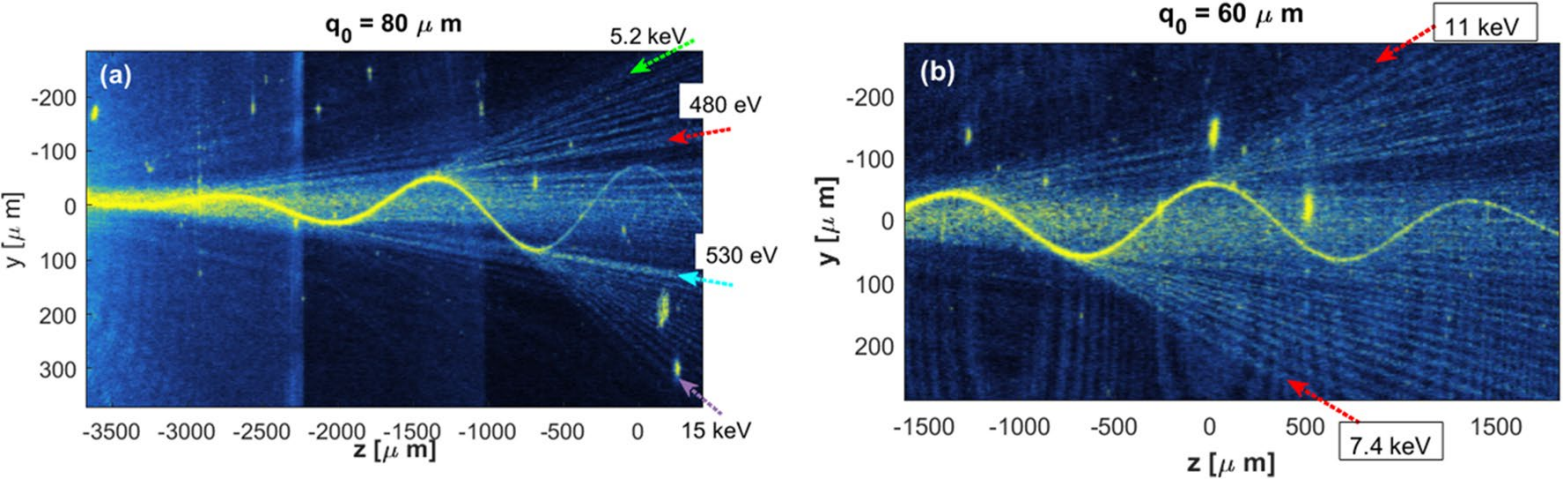
(**a**) waveguide with amplitude ${q}_{0}=80\mu m$, (**b**) Amplitude ${q}_{0}=60\mu m$. Both show leakage around the high curvature points and emitted rays which are reminiscent of the tunneled ATI electrons. In 2b. red arrows indicate the "ATI electrons" with the highest kinetic energies and are labeled with these energies. In 2a, green, red, cyan, and purple arrows indicate high and low energy "ATI electrons" with reference for more detailed analysis in Figs. 3 and 4 and text below.

**Figure 3:**
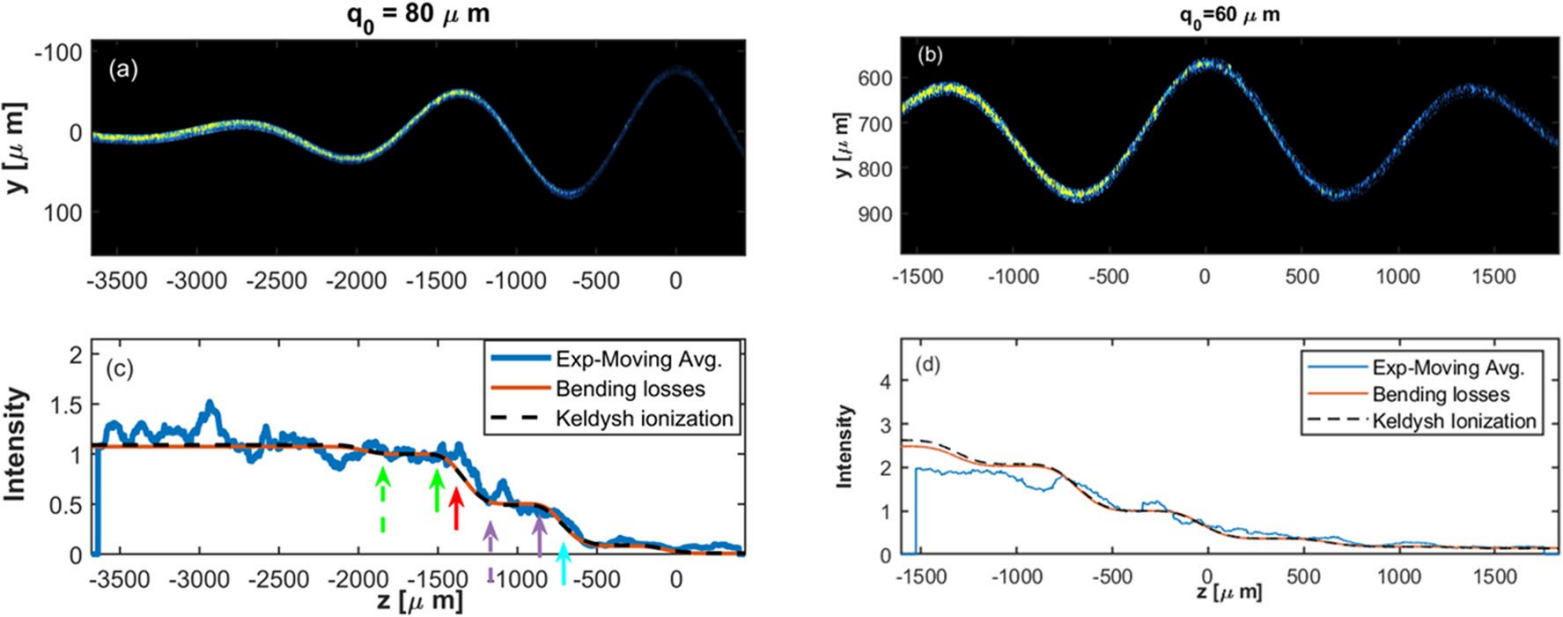
Shows the intensity inside the waveguides only, as a function of the z position. The left waveguide is the one with Amplitude ${q}_{0}=80\mathrm{\mu m}$, and the right waveguide is the one with ${q}_{0}=60\mathrm{\mu m}$. Figures 3c and 3d show the fitting of the integrated Keldysh and Marcuse rates to the experimental data. The Blue line represents a moving average over a range of $\Delta z=150\mathrm{\mu m}$. In 3c, the fitting curve and the experimental data are normalized to each other at $z=-1500\mathrm{\mu m}$. In Fig. 3d, they are normalized at $z=-200\mathrm{\mu m}$. Green, red, cyan, and purple arrows indicate the predicted emission "times" of the "ATI electrons" as appeared in Fig. 2 and in more details in Fig. 4.

**Figure 4:**
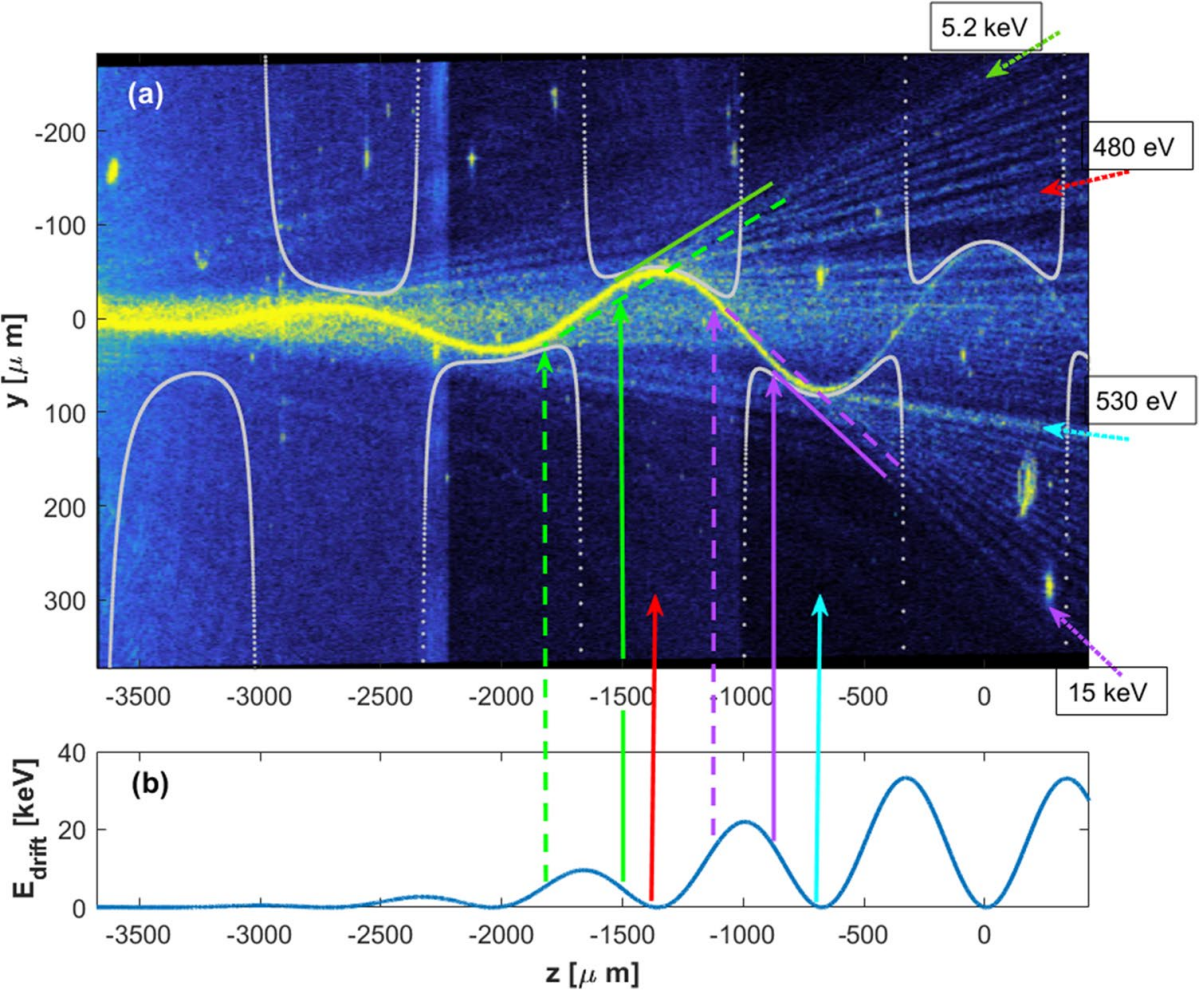
(**a**) as in Fig. 2a. waveguide with amplitude ${q}_{0}=80\mathrm{\mu m}$; leakages around the high curvature points and emitted rays which are reminiscent of the tunneled ATI electrons. Dotted green and purple arrows indicate the "ATI electrons" with the highest kinetic energies that are emerging from the vicinity of two high-curvature sections, and are labeled with their energies. Red and cyan dotted arrows indicate "ATI electrons" with low kinetic energies and are labeled with these energies. (**b**) calculated "drifting kinetic energy" as a function of the leakage position ("ionization time") according to the semiclassical model and with the assumption of zero velocity at the tunneling exit. The vertical arrows from 4b to 4a show the relations between the drifting energy and the position (instant) of ionization. Green and purple solid and dashed lines are parallel to the trajectories of the most energetic "ATI electrons" and are tangential to the waveguide at the two possible exit points. The gray curves show the calculated critical radius ${r}_{c}$ ,which is equivalent to the tunneling exit point.

Figure 2. shows two curved waveguides, one has an amplitude of $80\mu m$ (2a), the other has an amplitude of $60\mu m$ (2b). In the case depicted in (2a), we can see high bending losses around each high curvature point ($z\approx -2750, z\approx -2020,z\approx -1350, z\approx -700$) and emission of straight rays. A similar case happens with the ${q}_{0}=60\mu m$ waveguide around points $z\approx -1300, z\approx -680, z\approx 0$. The emitted straight rays shown in Fig. 2 are reminiscent of the ATI electrons. From the slope of those rays and the scaling given in Eq. (5) we have $d\widetilde{y}/d\widetilde{z}=d\widetilde{Q}/d\widetilde{t}={\text{v}}_{\text{drift}}/c$, thus, we can calculate the related drift kinetic energy of an electron that is moving along these rays. In Fig. 2b, the red arrows indicate the "ATI electrons" with the highest kinetic energies and are labeled with these energies. In Fig. 2a, the green, red, cyan, and purple dotted arrows indicate high and low energy "ATI electrons" with reference for more detailed analysis in Figs. 3 and 4 and text below. The green and purple dotted arrows indicate the cutoff kinetic energies of those “ATI electrons”.The strong background rays that are propagating along the central part are a mixture of leakage from the waveguide and uncoupled waves from the waveguide entrance.

Figure 3. shows the intensity inside the waveguides only, as a function of the z position. The lower panel shows the fitting of the integrated Keldysh and Marcuse rates to the experimental data. In our fitting, we multiply the Keldysh rate given in Eq. (7) by a factor of 1.3. We also found that the Marcuse rate (Eq. (6)) is overestimated by a factor of $\pi {e}^{2\gamma a}$, in accordance with^55^, and we dropped this factor. The solid green and purple arrows indicate the predicted emission positions ("times") of the most energetic "ATI electrons" as indicated with the dotted green and purple arrows in Fig. 2a. It seems that these cut-off “ATI electrons” are ionized just at the moment in which the ionization rate starts to be significant. “ATI electrons” with higher drifting energies are not observed because of the too low ionization rate. “ATI electrons” with lower drifting energies emerge at later times where the ionization rates are high (solid red and cyan arrows in Fig. 3c, dotted red and cyan arrows in Fig. 2a). The dashed red and purple arrows indicate a second option for the ionization “time” of the cut-off “ATI electrons” (see Fig. 4 and discussion below).

Figure 4a is the same as Fig. 2a, but with more details. In Fig. 4b we include also the calculated drifting energy of the "ATI electron" as a function of the "ionization time" (leakage z-position), calculated according to the classical mechanic's equation of motion $m\ddot{x}=-eE(t)$ with the initial conditions: $x\left({t}_{i}\right)=0, \dot{ x }\left({t}_{i}\right)=0, {{\text{where}} t}_{i}$ is the ionization time. The vertical arrows from 4b to 4a show the relations between the drifting energy and the position (instant) of ionization. The gray curves in Fig. 4a show the calculated critical radius ${r}_{c}$ ,which is equivalent to the tunneling exit point^51^.

To answer the question at what "time" the "electron" was ionized, we can trace back the "electron" straight trajectory toward the waveguide. The green and purple, solid and dashed lines in Fig. 4a are parallel to the trajectories of the most energetic "ATI electrons" and are tangential to the waveguide at the two possible "ionization" points according to the "electron drifting energy". The solid lines are tangential to the waveguide at the immediate ionization point from which the "electron" is immediately drifting away, while the dashed lines are tangential to the waveguide at the remoter ionization point from which the "electron" is first re-collides with the waveguide and only then is drifting away. If we try to take the two cut-off rays and trace back from which point are they emerged, we find that these trajectories are neither emerging from the nearer point nor from the remoter point. However, it is closer to the remoter exit point. It seems that the superposition of these two waves causes the interference pattern which appears as ray-like trajectories. For this reason, we cannot tell exactly from which point this ray is coming from. It demonstrates the limitation of the electron trajectories concept to adequately describe the tunneling ionization process which is a wave process in its nature. The ATI trajectory which is marked by the cyan dotted arrow and labeled with 530 eV (Fig. 4a) is another interesting feature to pay attention to. This trajectory seems thicker and brighter than its neighboring trajectories. If we check the critical radius ${r}_{c}$, we see that at the position $\text{z=-660 }\mu m$, the critical radius hits the waveguide wall and the barrier width becomes equal to zero. This situation is equivalent to the above-the-barrier ionization, thus, our simulator reflects nicely the transition from tunnel ionization to above-the-barrier ionization.

To conclude, we fabricated a curved waveguide with parameters relevant to simulate tunnel ionization of atoms under the action of a strong laser field. This simulation is based on the mathematical analogy between tunnel ionization and bending losses in optical waveguides^51^. In our curved waveguide, we observed an increased loss rate at locations with higher curvatures in accordance with theory. We also evaluated quantitively the losses inside the waveguide and compared them to the Keldysh tunnel ionization and the Marcuse bending losses equations. We found a good agreement with the Keldysh equation. For the Marcuse equation, we found that it is overestimating the leak rate by a factor of $\pi {e}^{2\gamma a}$ in accordance with^55^. Because of the high level of noise in our data, it is difficult to further differentiate between the two theories and we leave it for future work where we aim to have a lower noise level. From the high curvature areas, we observed straight rays that are analogous to the “ATI electrons”. While they have good agreement between the predicted drift energy of the “ATI electrons” and those energies in our simulator, we found in some cases that it is hard to precisely associate the ATI trajectories to a single tunneling exit point. This is probably because of the wave nature of tunneling where particle trajectories are only some simplifications. The observed ray like ATI trajectories are in fact an interference pattern from successive tunneling locations and it is difficult to associate the ATI to a single tunneling location just by tracing back the ATI trajectories. This work is a first demonstration of the abilities of such an optical quantum simulator. In the future, and in order to probe the tunneling process under the barrier, we plan to reduce the background noise, to improve the microscope resolution and to record the full wave information, i.e., amplitude and phase.

## Data Availability

The datasets used and/or analyzed during the current study are available from the corresponding author on reasonable request.
